# Changes in pressure distribution of the solar surface after a single trimming event are associated with external hoof measurements in the equine fore foot

**DOI:** 10.1111/evj.14463

**Published:** 2025-01-09

**Authors:** Sarah Seery, James Gardiner, Karl T. Bates, Gina Pinchbeck, Pete Clegg, Joanne L. Ireland, Peter I. Milner

**Affiliations:** ^1^ Department of Equine Clinical Science, Institute of Infection, Veterinary and Ecological Sciences University of Liverpool Neston UK; ^2^ Department of Sport and Exercise Sciences Manchester Metropolitan University Manchester UK; ^3^ Department of Musculoskeletal and Ageing Science, Institute of Life Course and Medical Sciences, The William Henry Duncan Building University of Liverpool Liverpool UK; ^4^ Department of Livestock and One Health, Institute of Infection, Veterinary and Ecological Sciences University of Liverpool Neston UK

**Keywords:** hoof, horse, pressure distribution, trimming

## Abstract

**Background:**

Trimming is critical for a functioning equine hoof. Pressure distribution provides information on loading; however, information on the effects of trimming on pressure distribution is lacking.

**Objectives:**

To describe the pressure changes of equine fore feet following trimming.

**Study design:**

Cross‐sectional cohort study.

**Methods:**

Fifty sound horses were recruited. Eighteen external hoof measures of the dorsal, lateral, medial and solar aspects were obtained before and after trimming from 94 fore feet. Horses were walked over a pressure mat before and after trimming and pressure maps of the solar surface created. Percentage change in hoof measures were assessed. Factors associated with an increase in pressure in the frog region after trimming were entered into a forward likelihood ratio logistic regression model. Odd ratios (ORs) with 95% confidence intervals (CI) and area under the curve receiver operator characteristics (AUROC) were calculated. Sensitivity and specificity were calculated at a cut‐off value of *p* = 0.5.

**Results:**

Trimming resulted in a significant increase in pressure, topographically mapped to the frog region, in 12/94 (13% 95% CI 6; 20) feet. Percentage difference in bearing border length (OR 0.66 95% CI 0.51; 0.86), heel buttress to centre of pressure distance (OR 1.30 95% CI 1.10; 1.53), heel angle (lateral side) (OR 1.11 95% CI 1.04; 1.19) and heel length (medial side) (OR 0.92 95% CI 0.85; 0.99) were retained in the final model associated with increased pressure in the frog region following trimming. AUROC was excellent (0.94 95% CI 0.88; 0.99) with fair sensitivity (58% [95% CI 50; 66]) and excellent specificity (98% [95% CI 78; 118]).

**Main limitations:**

Subjective lameness exam; horse velocity unmeasured.

**Conclusions:**

Measuring pressure changes over the solar surface of the equine fore foot after trimming identified that an increased pressure in the frog region was linked to specific changes in hoof shape.

## 1 INTRODUCTION

An important influence on hoof shape and loading in the horse is via the intervention of farriers and other hoof care professionals. Historical texts tend to focus on foot trimming as a method of achieving balance through symmetry.^1^, ^2^ Conventional farriery teaching has been based on these ideals although it is increasingly accepted that dynamic foot balance adapted to the individual horse's needs should be considered rather than a one‐size fits all approach to footcare.^3^, ^4^ Differences in foot shape between feet may result in abnormal loading patterns^5^ and could be linked to lower limb pathology and lameness^6^ although cause and effect has not been clearly demonstrated.^7^, ^8^

Research into hoof loading patterns in the horse has been predominately centred around the use of force and pressure plates to measure peak forces and dynamic loading under experimental and clinical conditions.^9^, ^10^, ^11^, ^12^ Some studies have considered the effect of hoof care interventions on loading patterns but the link between hoof shape and loading has not been fully established.^13^, ^14^, ^15^ Originally developed to assess changes in regional blood flow during functional brain imaging, statistical parametric mapping (SPM) uses pixel intensity changes to reflect organ function.^16^ SPM has been adopted for use in pedobarography^17^ and applied to areas such as substrate compliance, plantar pressure measurements and footprint depth in humans and ancient hominids.^18^, ^19^, ^20^ Compared with previous studies which report peak contact pressures in the equine hoof^14^, ^15^ or analyse defined regions (e.g., toe versus heel or medial versus lateral)^11^, ^12^, ^21^ pedobarographic SPM (pSPM) can provide topographical mapping through a continuous statistical field. This may be advantageous over other studies where although the magnitude of pressure patterns after an intervention may be determined (e.g., a reduction in total vertical pressure in the toe region was noted after shoeing with a shoe with wide toe)^11^ pSPM can offer an approach to analyse pressure patterns across the whole surface of the hoof. Despite its usefulness in studying intra‐subject geometry to the authors' knowledge, pSPM has not been used in equids.

The aims of this study were to describe pressure distribution of the solar surface of the equine fore foot before and after a single trimming event utilising pSPM and to identify whether these findings are associated with specific external hoof measurements. The main hypothesis was that pressure distribution maps would detail changes in loading before and after a single trimming event and through the application of pSPM these changes would be topographically mapped to specific regions of the foot. The second hypothesis was that specific external hoof measurements would be related to these changes.

## 2 MATERIALS AND METHODS

### 2.1 Data collection

Ten Worshipful Company of Farriers (WCF)‐registered hoof care professionals were recruited from the North‐West of England and North Wales, UK. The sample group of horses was recruited by convenience sampling through participating hoof care professionals between June 2017 and June 2019. Inclusion criteria were that horses were free of lameness (assessed at trot in a straight line on a hard surface by an RCVS‐registered veterinary surgeon at recruitment) and ridden at least once every 2 weeks.

**FIGURE 1:**
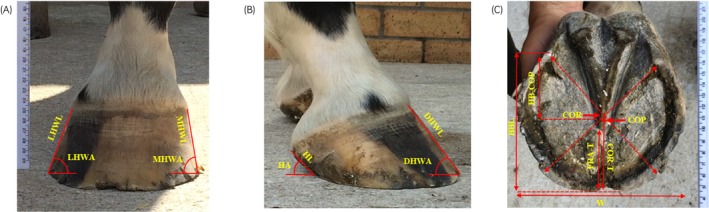
Digital photos of the (A) dorsal, (B) lateral and (C) solar aspects of the front foot with hoof measurements including LHWL (lateral hoof wall length), MHWL (medial hoof wall length), LHWA (lateral hoof wall angle), MHWA (medial hoof wall angle), DHWL (dorsal hoof wall length), DHWA (dorsal hoof wall angle), HL (heel length), HA (heel angle), W (width), BBL (bearing border length), COP (centre of pressure), COR (centre of rotation), COR‐T (centre of rotation to toe), HB‐COP (heel buttress to centre of pressure) and FRA‐T (frog apex to toe). Dashed lines (*without arrows*) indicate reference lines for the margins of the heel buttress and toe whereas dashed line (*with arrows*) indicate reference lines from the heel buttress to the opposite breakover point to indicate COR at their intersection. COP was measured as a point approximately 9.5 mm caudal to the apex of the frog. DHWL, DHWA, HL and HA were measured from both lateral and medial aspects.

Digital images (using a smartphone camera with a minimum of 8 megapixels) of left and right fore feet from the dorsal, lateral, medial and solar aspects of each fore foot were obtained by the same operator before and after a single trimming event (Figure 1). Dorsal images were centred on the midline of the hoof approximately 2 cm below the coronary band, lateral and medial images centred halfway between the dorsal hoof wall and heel approximately 1 cm below the coronary band, and solar images centred on the point of the frog. Images included a measurement scale at the time of image acquisition and horse details were anonymised. Eighteen external hoof measurements were calculated using ImageJ version 1.52a software (ImageJ, U.S. National Institutes of Health, Bethesda, MD, USA).^22^ Hoof measurements included lateral hoof wall length (LHWL, cm), medial hoof wall length (MHWL, cm), lateral hoof wall angle (LHWA, ϴ), medial hoof wall angle (MHWA, ϴ), dorsal hoof wall length (DHWL, cm), dorsal hoof wall angle (DHWA, ϴ), heel length (HL, cm), heel angle (HA, ϴ), width (W, cm), bearing border length (BBL, cm), centre of pressure to centre of rotation (COP‐COR), centre of rotation to toe (COR‐T, cm), heel buttress to centre of pressure (HBUT‐COP, cm) and frog apex to toe (FRA‐T, cm), adapted from Caldwell et al.^4^ DHWL, DHWA, HL and HA were measured from both lateral and medial aspects.

Horses were led by an experienced handler (usually the horse owner/caretaker or lead author SS) over a commercially available pressure mat (Matscan XL, Biosense Medical). Calibration of the pressure mat was performed prior to data acquisition, adapted from Oosterlinck et al.,^23^ involving measuring the mass of a horse with one foot stood on a weighbridge before calibrating the mat against that same horse with the same foot stood on the mat. This allowed the mat to calibrate against a relevant mass such that no or very few sensels (single element sensors within an array of elements) became saturated during data collection. To confirm this, pilot data were used to compare raw and smooth data sets of the 10 largest horses within the study which demonstrated no significant ceiling effect due to size. Recalibration was undertaken after every five uses to avoid sensor drift. Horses were walked over a flat concrete (or equivalent) surface with at least 5 m lead in prior to contacting the mat. A 3 mm rubber mat (present during both calibration and data collection) was placed over the pressure mat, following recommendation by the manufacturer, to reduce the risk of damage to the sensels. Video analysis was used to trigger data capture (EasyCam, Microsoft) and to identify correct foot placement. A minimum of four valid strikes per forelimb (where the whole fore foot is represented by the sensels) were recorded for each horse, before and after trimming for acceptable reliability.^24^ Incomplete strikes or strikes where the horse was deemed to have walked at an incorrect pace (too fast/too slow) were excluded from analysis.^25^

### 2.2 Pressure data processing and analysis

Pressure mat data were processed and analysed using custom‐written code, as well as functions from the pedobarographic statistical mapping (pSPM) package within MATLAB (The MathWorks, Inc.). Peak pressure prints were separated and assigned as left or right fore before the right fore prints were mirrored to allow comparison with left prints. All prints were then rotated and aligned to “register” prints to a common orientation. Once prints had been registered, comparison between conditions were performed (i.e., before and after trimming) using two‐sample Students *t*‐tests within pSPM methodology.^17^, ^18^, ^19^

### 2.3 Statistical analysis of non‐pressure data

Statistical analysis was performed using SPSS statistical software, version 29 for Windows (IBM). Each hoof measurement was calculated as a percentage difference before and after trimming to remove the effect of horse size on hoof measurement values.^26^ Distribution of data was assessed using visual inspection of histograms, Q–Q plots, Kolmogorov–Smirnov and Shapiro–Wilk tests for Normality and Levene's test for equality of variance. Student's *t*‐test or Mann–Whitney *U* test was used for univariable analysis. An adjusted *p*‐value of *p* < 0.05/18 = 0.003 was used for hoof measurements using Bonferroni correction. Correlations between explanatory variables were assessed using either Pearson's or Spearman rank correlation depending on data distribution and the most plausible variable was selected where variables were highly correlated (>0.8). Explanatory hoof measurement variables with univariable *p*‐values of <0.2 were entered in a stepwise fashion into a forward likelihood ratio logistic regression model for increased frog pressure after trimming and retained if they significantly improved the fit (*p* < 0.05). Hosmer–Lemeshow goodness of fit test statistic was used to assess the fitness of the model. Discrimination of the model was determined by calculating the area under curve receiver operator characteristic (AUROC) value (95% CI) and sensitivity and specificity were also calculated for the model at cut‐off value *p* = 0.5. Data are presented as mean (95% CI) or median (IQR).

## 3 RESULTS

### 3.1 Descriptive data

**FIGURE 2:**
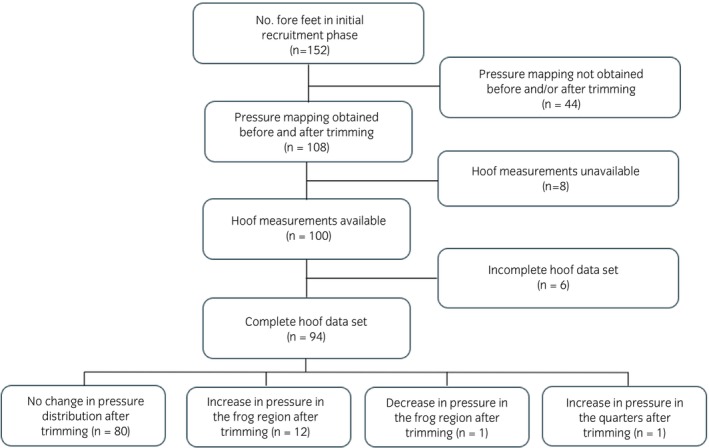
Flow chart illustrating number of fore feet having both complete hoof data set and pressure distribution maps before and after trimming for inclusion in the final analysis following initial recruitment.

Complete hoof measurement data and pSPM data were available for 94 front feet from 50 horses (Figure 2). There were 36 geldings and 14 mares. Mean age was 11.6 years (95% CI 10.4; 12.9). Breed types included warmblood/warmbloodX (WB/WBx) (*n* = 13), Thoroughbred/ThoroughbredX (TB/TBx) (*n* = 11), Irish Draught/Irish DraughtX (ID/IDx) (*n* = 9), Welsh/WelshX (*n* = 6), Arab/ArabX (*n* = 5), Connemara (*n* = 3), cob (*n* = 1), native pony (*n* = 1) and FriesianX (*n* = 1). Of the 10 farriers involved in the study, one farrier was responsible for trimming 21 hooves, two farriers trimmed 15 hooves each, two farriers trimmed 12 hooves each, one farrier trimmed seven hooves, one farrier trimmed six hooves and three farriers trimmed two hooves each. Median time from the previous trimming event was 6 weeks (IQR 5; 7).

### 3.2 Pressure distribution maps and hoof measurements after trimming

**FIGURE 3:**
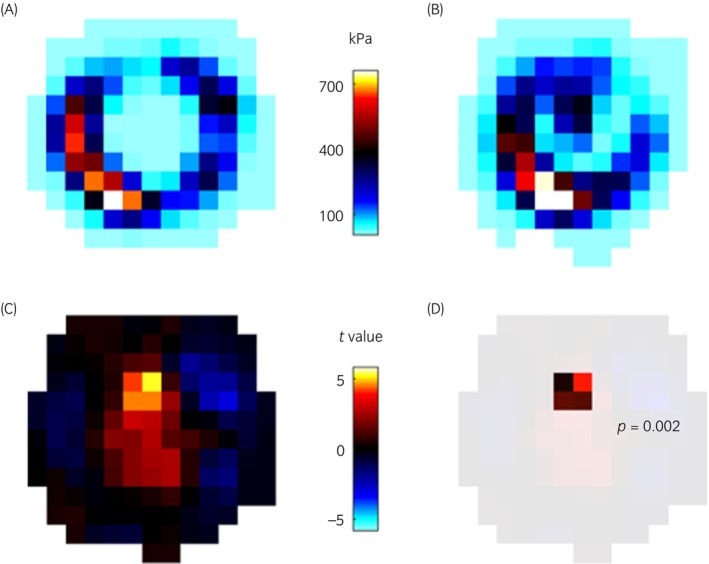
An example of increased pressure in the frog region after trimming. Individual prints were aligned, registered, and averaged for topographical comparison. Mean peak pressure data plots are shown (A) before (*n* = 19 strikes) and (B) after trimming (*n* = 14 strikes) of the left fore foot of horse no. 39. (C) Raw statistical parametric map constructed from pixel level *t*‐tests, with lighter regions indicating where the two means in (A) and (B) differ most. Blue pixels indicate lower pressure and yellow‐red pixels higher pressure in the after trimming mean record with (D) showing a cluster of four pixels mapping to the frog region that have a statistically significant difference in pressure (*p* = 0.002). Data in (A) and (B) are presented in kilopascals (kPa). Data in (C) and (D) are *t* values.

**FIGURE 4:**
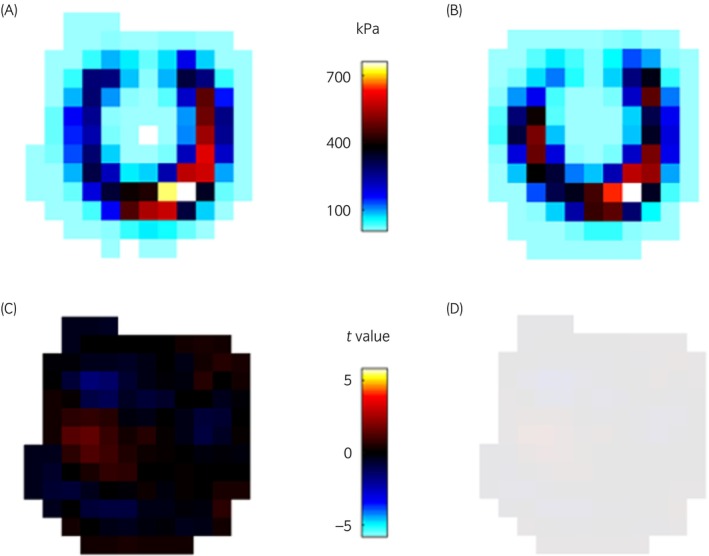
An example where no pressure change occurred after trimming. Individual prints were aligned, registered, and averaged for topographical comparison. Mean peak pressure data plots are shown (A) before (*n* = 14 strikes) and (B) after trimming (*n* = 14 strikes) of the right fore foot of horse no. 92. (C) Raw statistical parametric map constructed from pixel level *t*‐tests. (D) No significant differences were present in this sample after trimming. Data in (A) and (B) are presented in kilopascals (kPa). Data in (C) are *t* values.

After trimming there was a significant change in pressure in 14/94 (15% 95% CI 8; 22) fore feet of which 12/14 (86% 95% CI 70; 100) fore feet demonstrated a significant increase in pressure topographically mapped to the frog region (Figure 3). Of the remaining 2/14 (14% 95% CI −4; 32) fore feet, one demonstrated a significant reduction in pressure in the frog region and one demonstrated significant increases in pressure in the medial and lateral quarters of the sole after trimming. No significant difference in pressure distribution after trimming was recorded in 80/94 (85% 95% CI 78; 92) fore feet (Figure 4). Of the 12 fore feet with increased pressure in the frog region after trimming, 7/12 (58% 95% CI 30; 86) were accounted for by one farrier, 3/12 (25% 95% CI 0.5; 50) to another farrier and 1/12 (8% 95% CI −7; 24) each to two other farriers.

Table 1 shows univariable analysis of the percentage change of each hoof measurement before and after trimming. A percentage reduction in the distance between FRA‐T, DHWL (from the lateral side) and medial HL was associated with increased pressure in the frog region after trimming. A percentage increase in HA after trimming from both lateral and medial sides was also associated with an increase in pressure. Following correction for multiple comparison (Bonferroni‐adjusted *p* = 0.003), only an increase in HA after trimming (from the lateral side) remained significant.

**TABLE 1:** Univariable analysis of hoof measurements before and after trimming in 94 front feet.

Hoof measure	% difference after trimming (95% CI)		*p*‐value
	No increase in pressure in the frog region after trimming (*n* = 82)	Increase in pressure in the frog region after trimming (*n* = 12)	
Lateral hoof wall length (LHWL)	−5.1 (−6.7; −3.5)	−6.4 (−12.6; −0.2)	0.6
Lateral hoof wall angle (LHWA)	1.6 (0.8; 2.4)	2.6 (−0.7; 6.0)	0.4
Medial hoof wall length (MHWL)	−4.4 (−6.3; −2.6)	−7.1 (−14.8; −0.6)	0.3
Medial hoof wall angle (MHWA)	1.9 (−3.3; 7.0)^‡^	4.3 (0.4; 8.2)	0.1^†^
Dorsal hoof wall length (DHWL) (from lateral side)	−5.6 (−14.0; 2.9)^‡^	−11.2 (−14.6; −7.7)	0.007^†^
Dorsal hoof wall angle (DHWA) (from lateral side)	−2.9 (−4.1; −1.8)	−5.1 (−8.1; −2.1)	0.2
Heel length (HL) (from lateral side)	−5.7 (−22.4; 11.0)^‡^	−10.1 (−19.6; −0.7)	0.2^†^
Heel angle (HA) (from lateral side)	2.5 (−0.6; 5.5)	18.5 (6.8; 30.3)	**<0.001**
Dorsal heel wall length (DHWL) (from medial side)	−6.3 (−7.8; −4.9)	−10.6 (−14.8; −6.4)	0.04
Dorsal hoof wall angle (DHWA) (from medial side)	3.2 (2.1; 4.3)	2.3 (0.1; 4.6)	0.6
Heel wall (HL) (from medial side)	−7.2 (−27.1; 12.7)^‡^	−17.8 (−25.9; −9.8)	0.006^†^
Heel angle (HA) (from medial side)	0.002 (−3.8; 3.8)	6.8 (−11.9; 25.5)^‡^	0.02^†^
Width (W)	−2.8 (−8.6; 3.0)^‡^	−4.1 (−9.6; 1.3)	0.5^†^
Bearing border length (BBL)	−0.4 (−1.9; 1.1)	−2.5 (−8.6; 3.6)	0.4
Centre of pressure‐centre of rotation (COP‐COR)	13.5 (−25.6; 52.6)^‡^	51.3 (13.6; 89.0)^‡^	0.06^†^
Centre of rotation to toe (COR‐T)	0.1 (−8.3; 8.4)^‡^	−1.4 (−8.5; 5.7)	0.7^†^
Heel buttress to centre of pressure (HB‐COP)	2.2 (−0.2; 4.6)	8.3 (−2.3; 18.9)	0.1
Frog apex to toe (FRA‐T)	−5.4 (−19.3; −8.5)^‡^	−12.6 (−18.2; −7.0)	0.01^†^

*Note*: Values are represented as percentage change after trimming where a negative value indicates a percentage reduction after trimming and a positive value represents a percentage increase after trimming. Student's *t* tests were used for normally distributed and Mann–Whitney *U* tests^†^ for non‐normally distributed data, with significance assumed at *p* = 0.003 after Bonferroni correction. Data are presented as mean (95% CI) or median (IQR)^‡^. *p*‐values remaining significant after correction are highlighted in bold.

### 3.3 Prediction model for increased pressure in the frog region after trimming

**FIGURE 5:**
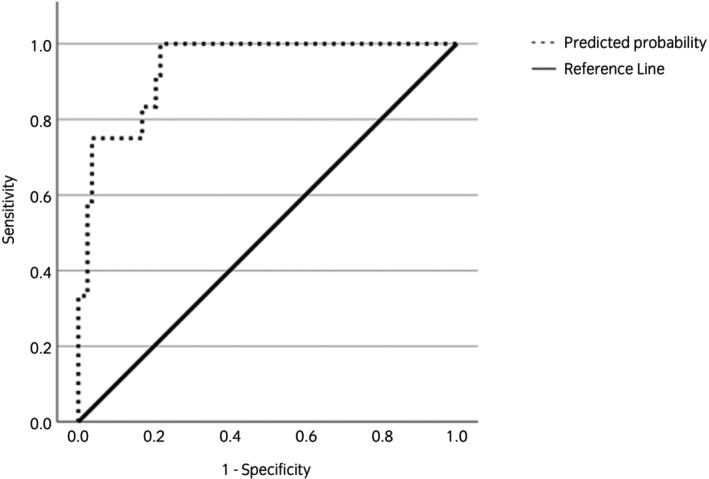
Receiver operator curve (ROC) for the final predictive model of increased frog pressure in the fore foot after trimming. Area under curve receiver operator characteristic (95% CI) for this was 0.94 (0.88; 0.99).

Table 2 shows the final multivariable model for the prediction of increased pressure in the frog region after trimming. Correlation coefficients between explanatory variables were all <0.8 and so all hoof measurement variables were entered into the model. Hoof measurement variables retained in the model were percentage differences after trimming for HA (from the lateral side), BBL, HB‐COP and medial HL. The *p*‐value for the Hosmer and Lemeshow test statistic was *p* = 0.4 showing adequate fit of the model. The AUROC for the final model was 0.94 (95% CI 0.88; 0.99) (Figure 5). Sensitivity and specificity of the model at a cut‐off value of *p* = 0.5 were 58% (95% CI 50; 66) and 98% (95% CI 78; 118), respectively.

**TABLE 2:** Final logistic regression model^a^ for increased pressure in the frog following trimming (*n* = 94 front feet).

Variable	Coefficient	Standard error	Adjusted odds ratio	95% confidence interval	Likelihood ratio *p*‐value
% difference bearing border length (BBL)	−0.41	0.13	0.66	(0.51; 0.86)	0.002
% difference heel buttress to centre of pressure (HB‐COP)	0.26	0.09	1.30	(1.10; 1.53)	0.002
% difference heel angle (HA) (lateral aspect)	0.10	0.34	1.11	(1.04; 1.19)	0.003
% difference heel length (HL) (medial aspect)	−0.09	0.04	0.92	(0.85; 0.99)	0.03
Intercept	−6.29	1.58			

^a^ Multivariable logistic regression equation, *x* = −6.29 + [(−0.41 × BBL) + (0.26 × HB‐COP) + (0.10 × HA (lat.)) + (−0.09 × HL (med.))] where predicted probability (*P*) = *e* ^*x*^/(1 + *e* ^*x*^).

## 4 DISCUSSION

The main aim of this study was to use pressure distribution mapping and compare the changes in shape and loading of the fore feet of sound horses after trimming. An increase in pressure mapped to the frog region after trimming was identified in 13% of front feet. This was significantly associated with an increase in lateral HA (univariable analysis) with reductions in BBL and medial HL and increases in the distance between HB‐COP and lateral HA being retained in the final predictive logistic regression model for increased pressure in the frog region. Increased pressure in the frog region could result in pain^27^ or lead to compensatory adaptation of the hoof thereby impacting the long‐term athletic function of the horse. By recognising the association with key hoof measures, modifications to mitigate this effect would be a beneficial means of preventative foot care.

From our results viewed from the solar aspect a shorter BBL and longer HB‐COP would equate to stretching (relative lengthening) of the frog which was highlighted by Caldwell et al.^4^ With a concurrent lowering of the medial heel length and a steeper lateral heel angle it could be envisaged that flattening of the sole and distortion of heels alters the loading pattern on the solar aspect of the foot. The concavity of the solar surface, through its dome shape, contributes to the distribution of load across the surface of the foot^28^ and flattening of the sole would likely compromise this role leading to an increase in pressure elsewhere such as the frog. Since the frog is integral to dissipating load during locomotion, increased pressure in this region could lead to a re‐distribution of load to the hoof wall resulting in wall compression and hoof deformation.^29^ If concurrent heel distortions were present, these could additionally result in changes in stress through the hoof capsule with implications on the structure of the hoof, such as at the laminar junction.^30^ Focal increases in pressure can also directly cause lameness.^27^ However, the effects of continued pressure in this region have not been clearly demonstrated, with both expansion and restriction of the heels reported during weight‐bearing,^31^, ^32^ although it must be highlighted these studies use external devices to increase pressure on the frog rather than measuring increased frog pressure per se. Nevertheless, protocols where trimming encourages engagement of frog with the ground surface through the belief of the frog pressure theory could in fact be detrimental and have been previously challenged by other authors.^33^

Many studies evaluate hoof shape from one or two planes^7^, ^8^ but it is clear from this study key information is gained from evaluation from all aspects of the foot, particularly the solar surface. Evaluation from the medial side as well as lateral is rarely reported and may provide laterality as seen here where the association of the heel length following trimming was only evident from the medial side. The use of percentage changes after trimming rather than reporting raw values was similar to the approach adopted by Kummer et al.^26^ This removes the effect of horse size and allows comparative measurements over a range of horse and pony breeds thereby making the findings universally applicable.

Image analysis for pressure distribution maps requires registration of foot‐strikes which can be demanding as pixel resolution is often rigid (and dependent on sensel number) a perfect overlap is rarely achieved. Despite this, the process uses foot geometry and therefore the overlap ratio of images is usually high (>80%). Once registered though the statistical analysis is relatively rapid and not affected by smoothing.^19^ Traditional approaches to pressure data often analyse single pressure values from zones (e.g., using quadrant analysis) or the entire print (e.g., maximum pressure). However, pSPM accounts for the fact that pressure is distributed continuously across the prints and therefore neighbouring pixels in the data are not independent.^17^ The methods ultimately allow for clusters of pixels on mean prints to be identified as significantly different between conditions with the magnitude of difference (*t* value) topographically mapped to the solar surface of the hoof.

A limitation of the present study was the relatively low sensel density of the mat used (0.3 sensels/cm^2^) and whether this would affect the sensitivity to detect change, for example, if there were an effect from a change in the overall size of the solar surface following trimming. The extent of change of the solar surface occurring after trimming (as estimated by change in BBL and width), however, appeared relatively low (0.5%–2.5% and 3%–4%, respectively) and unlikely to have had a measurable effect. Ideally, a large mat with a higher sensel density would have improved resolution to confirm this but was not available at the time of the study. Despite this, significant differences were demonstrated after trimming, reflected by the magnitude of the change (*t* value). Based on the resolution though, these changes could only be described to a region (i.e., the frog) rather than detailing smaller‐scale changes within a region, as may be achievable, for example, between individual digits in a human foot using a high sensel density mat.

Many studies state that between 4 and 12 pressure records will provide valid and reliable interpretation of pressure distribution in humans.^24^, ^34^ In contrast McClymont et al. showed that the range in pixel‐level mean square error at low subsamples (<50) was 25%–75% higher than that of full datasets consisting of >500 pressure records per subject. They subsequently argued that, at least in human studies evaluating plantar pressure, smaller sample sizes (*n* < 20 records) may capture a relatively low proportion of variance evident in larger data sets which may not accurately reflect the true population mean.^35^ Acquiring >500 pressure records per subject in the horse would represent a significant logistical challenge but there should be consideration of the range of data when sampling relatively low numbers of events. In equine studies, five valid measurements have been considered acceptable^11^, ^12^, ^23^ with low variability of data reported.^36^ In the present study a mean of 13.7 (95% CI 12.9, 14.6) strikes per foot was achieved. Horses were walked over the pressure mat and although velocity was not directly measured it was subjectively assessed by the lead author. There may have been variation in velocity at walk during data acquisition, although this was not noted, nor was there any incidence of foot‐soreness or lameness reported after trimming. Van Heel et al. found a small but significant reduction in velocity at trot after trimming although this did not affect vertical ground reaction force or hoof‐unrollment pattern.^3^ An additional challenge when studying large animals (400 kg+) is the potential ceiling of data through sensel saturation. We found that calibration using humans was insufficient for the present study, so adapted a calibration method from Oosterlinck et al.,^23^ was used and validated, as described in the methods.

Convenience sampling was used to recruit farriers/hoof care professionals and horse owners/carers in the North‐West of England and North Wales. This may have biased the sample obtained towards those farriers/hoof care professionals interested in research and similarly to those horse owners/carers who are more engaged with horse health and foot care.^37^ Horses were screened for lameness by the lead author (SS) with lame horses excluded from the study. It is to be acknowledged that although horses were deemed sound through subjective analysis, objective measures of lameness may have revealed subtle lameness.^38^ Additionally the potential impact of previous episodes of lameness reported by the owner/carer was not taken into consideration in this study as the horses were sound at the time of evaluation. Although potentially influencing hoof loading patterns, conformation (e.g., toe‐in or toe‐out) was not assessed because of its subjective nature since the primary focus of this study was on objective measures before and after trimming. Through the assessment of the foot from all planes, these objective data should reasonably encompass common foot types.^39^

Following domestication and the use of horses historically for manual work and, more recently, pleasure and competitive purposes, the trimming of horses' hooves has been an essential requirement for the management and maintenance of foot shape and function. Currently, there are clear guidelines and standards for trimming horses' hooves in the UK through the Farriers Registration Council leading to membership in the Worshipful Company of Farriers (WCF). These recognised standards protect the horse and horse owners/carers. All farriers and hoof care professionals recruited to this study were members of the WCF and therefore have attained the required standard for hoof care in the United Kingdom. However, it was clear from the present study that there was a farrier effect associated with an increase in pressure in the frog region after trimming, with one farrier accounting for 58% of those fore feet. Despite the standardised training of farriers there clearly exists variation in the application of trimming protocols. This has been described by Kummer et al., where difference between farriers and within farriers on consecutive trimmings was demonstrated.^40^ Handedness of the farrier has also been highlighted as a potential reason for differences in trim patterns,^41^ although this was not determined in the present study. To account for farrier difference, it was important to quantify what changes in objective hoof measures were associated with an increase in pressure in the frog region after trimming. In doing so, this could provide feedback to modify trimming and allows the prediction model to be applicable outside of the farrier cohort used.

In conclusion, this study demonstrated a link between hoof shape and function by documenting the effect of trimming on pressure distribution and identifying key changes related to areas of increased pressure. Specifically, to mitigate an increase pressure in the frog region of the fore foot after trimming, hoof care professionals should avoid reducing BBL excessively along with medial HL and prevent an overlengthening of the distance between HB and COP, while paying particular attention to avoiding marked increases in lateral HA. The approach detailed could also be used in prospective studies to identify at‐risk cases for potential foot‐related pain thereby providing a quantifiable means to modify foot shape as a preventive measure. Future research would be through longitudinal studies relating foot shape to outcome as well as interventional studies involving corrective farriery and surgery.^42^, ^43^

## FUNDING INFORMATION

This study was supported by the Horse Trust, reference: G3016.

## CONFLICT OF INTEREST STATEMENT

The author declares no conflicts of interest.

## AUTHOR CONTRIBUTIONS

**Sarah Seery:** Methodology; software; writing – review and editing; writing – original draft; investigation; formal analysis; data curation; resources; validation. **James Gardiner:** Methodology; software; data curation; formal analysis; writing – review and editing. **Karl T. Bates:** Writing – review and editing; software; data curation; formal analysis; methodology. **Gina Pinchbeck:** Writing – review and editing; supervision. **Pete Clegg:** Supervision. **Joanne L. Ireland:** Writing – review and editing; supervision. **Peter I. Milner:** Conceptualization; methodology; supervision; formal analysis; project administration; funding acquisition; writing – original draft; writing – review and editing; visualization; validation.

## DATA INTEGRITY STATEMENT

Peter I. Milner had full access to all the data in the study and take responsibility for the integrity of the data and the accuracy of the data analysis.

## ETHICAL ANIMAL RESEARCH

Project approval via institutional ethical review (VREC538; approval date 28/04/2017).

## INFORMED CONSENT

Owner consent was obtained.

## PEER REVIEW

The peer review history for this article is available at https://www.webofscience.com/api/gateway/wos/peer-review/10.1111/evj.14463.

## Data Availability

The data that support the findings of this study are openly available at http://doi.org/10.17638/datacat.liverpool.ac.uk/2765.
